# Associations of physical fitness with sleep quality and anxiety Among university students: a cross-sectional regression analysis

**DOI:** 10.3389/fspor.2026.1795033

**Published:** 2026-04-22

**Authors:** Gang Xu, Xia Li, Guangwen Song, Hongli Yu

**Affiliations:** School of Physical Education, Sichuan University of Science & Engineering, Zigong, China

**Keywords:** physical fitness, sleep quality, anxiety, regression analysis, university students

## Abstract

**Background:**

Poor sleep quality and anxiety are prevalent among university students and pose growing public health concerns. Physical fitness is often assumed to be associated with better sleep and mental health outcomes; however, evidence based on objectively measured physical fitness in non-clinical student populations remains limited and inconsistent.

**Objective:**

This study aimed to examine the associations between multiple objectively assessed physical fitness components and sleep quality as well as anxiety outcomes among undergraduate students.

**Methods:**

A total of 50 undergraduate students (mean age: 20.3 ± 1.1 years; 26 males, 24 females) enrolled in general physical education courses participated in this cross-sectional study. Physical fitness was assessed using standardized tests, including vital capacity, 50-m sprint, standing long jump, and sit-and-reach. Sleep quality was evaluated using the Pittsburgh Sleep Quality Index (PSQI), and anxiety symptoms were assessed using the anxiety subscale of the Hospital Anxiety and Depression Scale (HADS-A). Outcomes were analyzed as both continuous scores and categorical risk indicators (sleep disturbance: PSQI > 5; elevated anxiety: HADS-A ≥ 8). Multivariable linear and binary logistic regression analyses were performed, adjusting for age and sex.

**Results:**

After adjustment for age and sex, none of the physical fitness measures were significantly associated with PSQI global score or HADS-A score in linear regression analyses (all *p* > 0.05). Similarly, binary logistic regression analyses revealed no significant associations between physical fitness components and sleep disturbance or elevated anxiety symptoms (all *p* > 0.05). These null findings were consistent across different fitness domains and analytical approaches.

**Conclusions:**

Objectively measured physical fitness was not significantly associated with sleep quality or anxiety outcomes among non-clinical undergraduate students. These findings suggest that general physical fitness alone may have limited explanatory value for sleep and anxiety in university populations and highlight the importance of considering broader psychosocial and behavioral factors when addressing student mental health.

## 1 Introduction

Sleep quality and mental health have become major public health problems faced by college students around the world. Entering the college stage is usually accompanied by increased academic pressure, irregular work schedule, prolonged exposure time of electronic screens and increased psychosocial stress, which may have an adverse impact on sleep patterns and mental health (1, 2). Epidemiological studies continue to show that the prevalence of poor sleep quality and anxiety symptoms among undergraduates is high, emphasizing the need to identify changeable factors to improve the healthy outcome of the population (3, 4). Conceptually, sleep is embedded within a broader lifestyle system in which physical activity, sedentary behavior and screen exposure, and psychosocial stress co-occur and may jointly influence mental functioning. Against this backdrop, clarifying the role of physical activity and objective physical fitness in relation to sleep and anxiety is essential for developing more integrated and effective campus health interventions.

Physical activity and physical fitness are often regarded as protective factors that promote sleep and mental health at the same time. Growing research evidence shows that regular physical activity and structured exercise intervention can improve sleep quality and reduce anxiety and depression symptoms, which has a positive effect in different age groups (5, 6). Therefore, promoting physical fitness has become the core content of the health promotion strategy of colleges and universities. However, most of the existing evidence comes from interventional research, or is highly dependent on self-reported physical activity indicators, which are susceptible to recall bias and may not accurately reflect the real physical ability of individuals (7).

It should be noted that physical activity behavior and physical ability are both related and different concepts. Physical activity reflects more behavioral participation, while physical fitness reflects the physiological ability of individuals in many fields such as cardiopulmonary endurance, muscle strength, speed and flexibility (8). Therefore, objective physical fitness assessment may provide more supplementary information than the level of self-reported activities. Nevertheless, at present, there are relatively few studies on the systematic exploration of the relationship between objective measurement of physical fitness components and sleep or anxiety outcomes for non-clinical college students.

The results of the existing observational research on the relationship between young people's groups and mental health are inconsistent. Some studies found that there was a certain correlation between higher physical fitness levels and better psychological outcomes, while other studies observed that the relationship was weaker or not significant after controlling demographic and situational factors (9, 10). These inconsistent results suggest that the relationship between physical ability and mental health outcomes may be affected by situational factors and depends on population characteristics, baseline health status and analysis methods. In particular, it is not clear whether general physical fitness is enough to explain the differences in sleep quality and anxiety symptoms among healthy college students.

In addition, a large number of studies in the past mainly focused on a single outcome, or analyzed only based on continuous symptom scores, which may ignore the clinically meaningful threshold of poor sleep quality or anxiety elevation. Including continuous and classified outcomes in the analysis at the same time may help to understand the relationship between physical fitness and mental health indicators more comprehensively (11). To make up for these shortcomings of methodology, it is of great significance to clarify the role of physical fitness in students’ health and provide evidence-based basis for the practice of health promotion in colleges and universities.

Therefore, this study aims to explore the relationship between the physical fitness components of a variety of objective evaluations, including cardiopulmonary function, speed, muscle explosive force and flexibility, and the sleep quality and anxiety outcomes of undergraduates. Sleep quality and anxiety are measured by a verified scale, and analyzed by continuous scores and classified risk indicators respectively. By focusing on non-clinical college students and adopting objective physical fitness measurement, this study aims to clarify the degree of correlation between general physical fitness and sleep quality and anxiety symptoms in daily academic situations.

## 2 Materials and methods

### 2.1 Research design and research objects

This cross-sectional research is carried out on undergraduates of the public physical education course of Sichuan University of Science & Engineering (SUSE) in China. A total of 50 undergraduates voluntarily participated in this study (average age 20.3 ± 1.1 years old), including 26 males and 24 females. Subjects are recruited through course notifications and classroom lectures.

Based on the statistical analysis based on continuous outcome variables, this study uses G*Power software (version 3.1) to evaluate the sufficiency of the sample size. Based on the multivariate linear regression fixed model (R^2^ deviation from zero value), bilateral significance level of 0.05, statistical efficiency 80% and expected medium effect (Cohen's f^2^ = 0.15), and under a parsimonious modeling strategy with a limited number of predictors, the existing sample size is considered to be sufficient to detect associations of practical relevance (12, 13). This method is usually used in cross-sectional exploratory studies of college students to explore the relationship between physical ability and mental health outcomes (14, 15).

All participants signed a written informed consent form before the start of the study. This research program has been reviewed and approved by the Ethics Committee of the Fourth People's Hospital in Zigong City, China (ethical approval number: EC-2025-081), and all research procedures are in accordance with the Helsinki Declaration.

### 2.2 Participants and eligibility

Full-time undergraduate students were recruited from SUSE.

Inclusion criteria were: (1) aged 18–25 years; (2) enrolled as full-time undergraduates; (3) able to complete the physical fitness assessment and questionnaires; and (4) provided informed consent.

Exclusion criteria were revised to match the information actually collected in this study: (1) acute illness or injury at the time of assessment that prevented completion of physical fitness testing; and (2) incomplete key outcome data (PSQI or HADS-A) and missing key physical fitness test data.

Because this study did not collect information on psychiatric diagnoses or treatment history, diagnostic-based exclusion criteria was removed and instead clarified participant characterization as follows: In the present study, “non-clinical” refers to a non-treatment-seeking, community-based undergraduate sample recruited from the general student population rather than through clinical services. No clinical diagnostic interviews were conducted; therefore, questionnaire scores reflect self-reported symptom levels rather than clinical diagnoses.

### 2.3 Study setting

This cross-sectional study was conducted at Sichuan University of Science & Engineering, Zigong City, China between August 2024 and December 2024. All physical fitness tests and questionnaire assessments were administered on-site by trained staff following standardized procedures.

### 2.4 Physical fitness measurement

A Physical fitness assessment adopts the standardized on-site test method widely used in the physical health test of Chinese colleges and universities, and refers to previous literature reports (16, 17). All measurements are carried out by trained physical education teachers according to the unified test process.

The physical composition of the assessment includes:

**Vital capacity:** Use a calibrated pulmonometer to measure lung function, and the results are recorded in milliliters (mL). Lung capacity is regarded as an effective indicator of young people's respiratory and cardiopulmonary function (18).
**50-meter sprint:** use electronic timing equipment to measure the sprint performance and record the completion time (seconds).
**Standing long jump:** evaluate the explosive force of the lower limbs by measuring the jumping distance (cm).
**sit-and-reach performance:** the standardized sitting forward bending test box is used to evaluate the flexibility, and the results are recorded in centimeters (cm).

These on-site physical fitness tests have been proven to have good confidence and validity among college students.

### 2.5 Sleep quality assessment

Sleep quality is evaluated by the Pittsburgh Sleep Quality Index (PSQI), which is a widely used empirical self-assessment questionnaire, which can be used to evaluate the subjective sleep quality of clinical and non-clinical people (19). PSQI contains a total of 19 entries, which can calculate the total score of 0–21. The higher the total score, the worse the sleep quality.

Participants completed the questionnaire independently in a quiet classroom under the supervision of researchers. According to the established standards, PSQI total score greater than 5 is used to define sleep disorders in classification analysis (20).

### 2.6 Anxiety assessment

Anxiety symptoms are evaluated by the Hospital Anxiety and Depression Scale (HADS), which is a self-assessment tool commonly used to detect anxiety and depression symptoms in non-psychiatric people (21). There are 14 entries in the scale, including 7 anxiety component table entries (HADS-A) and 7 depression component table entries.

In this study, only the HADS-A component table was used to evaluate anxiety symptoms. Each entry adopts a 4-level score (0–3 points), and the total anxiety score ranges from 0 to 21 points. The higher the total score, the more serious the anxiety symptoms. Based on the existing verification research, HADS-A ≥ 8 was used in the classification analysis to define the increased anxiety level (22).

To ensure data accuracy, questionnaire scoring was double-checked according to the original scoring manuals, and total scores were verified to fall within the valid ranges (PSQI: 0–21; HADS-A: 0–21).

### 2.7 Statistical analysis

All statistical analysis is done with common statistical software. Continuous variables are expressed as mean ± standard deviation (SD), and classified variables are expressed as frequency and percentage.

The association between physical fitness components and continuous mental health outcomes (PSQI total score and HADS-A score) is analyzed by multivariate linear regression model, and the regression coefficient (*β*), 95% confidence interval (CI) and p value are reported.

For the dichotomized outcome variables, including poor sleep (PSQI > 5) and elevated anxiety (HADS-A ≥ 8), exploratory logistic regression was used to estimate odds ratios (ORs) and corresponding 95% CIs. These dichotomized analyses were conducted to enhance clinical interpretability and comparability with prior literature. Given the relatively small sample size and the high prevalence of the outcomes, the dichotomized models were considered secondary/exploratory and were interpreted cautiously. To reduce the risk of overfitting, the number of independent variables included in each model was restricted, consistent with established methodological recommendations (23).

Given the sample size and to minimize overfitting, each physical fitness component was examined in a separate regression model (i.e., fitness predictors were not entered simultaneously).

All regression models adjust age and gender as potential confounders. Model diagnostics were performed for all regression analyses: multicollinearity was assessed using variance inflation factors (VIF), residual plots were inspected for linear regression assumptions, and logistic regression models were evaluated using standard goodness-of-fit and influence diagnostics. The statistical significance level is set to *p* < 0.05 (two-tailed).

## 3 Results

### 3.1 Baseline characteristics of research objects

A total of 50 undergraduates were included in the analysis. The average age of the study subjects was 20.3 ± 1.1 years old, including 26 males (52.0%) in Table 1. In terms of physical fitness indicators, the averages of lung capacity, 50-meter sprint, standing long jump and sitting forward flexion are 3966.70 ± 2911.62 mL, 8.52 ± 1.18 s, 198.86 ± 32.31 cm and 14.41 ± 6. 28 cm.

**Table 1:** Baseline characteristics of the study participants (*n* = 50).

Variable	Total (*n* = 50)
Demographic characteristics	
Age, years	20.3 ± 1.1
Male, n (%)	26 (52.0)
Physical fitness measures	
Vital capacity (mL)	3966.70 ± 2911.62
50-m sprint (s)	8.52 ± 1.18
Standing long jump (cm)	198.86 ± 32.31
Sit-and-reach (cm)	14.41 ± 6.28
Sleep-related measures	
PSQI global score	13.22 ± 7.72
Sleep disturbance (PSQI > 5), n (%)	38 (76%)
Anxiety-related measures	
HADS-A score	17.28 ± 7.41
Elevated anxiety symptoms (HADS-A ≥ 8), n (%)	45 (90%)

Continuous variables are presented as mean ± SD; categorical variables are presented as n (%).

In terms of sleep-related outcomes, the average PSQI total score was 13.22 ± 7.72, and a total of 38 participants (76%) met the sleep disorder determination criteria (PSQI > 5). In terms of anxiety-related outcomes, the average HADS-A score was 17.28 ± 7.41, and a total of 45 participants (90%) were classified as increased anxiety levels (HADS-A ≥ 8). All PSQI and HADS-A total scores were within their valid ranges, and no out-of-range values were detected after verification.

### 3.2 Associations between physical fitness indicators and sleep quality

The correlation between physical fitness indicators and sleep quality evaluated by PSQI total score is shown in Table 2. After adjusting the age and gender, no significant correlation was found between the physical fitness indicators and the total PSQI score (all *p* > 0.05). There is a negative but not significant correlation between lung capacity and PSQI score (*β* = −0.139, 95% CI: −0.001 ∼ 30.000, *p* = 0.361). Similarly, there is no significant correlation between 50-meter sprint performance and sleep quality (*β* = 0.086, 95% CI: −3.044 ∼ 4.165, *p* = 0.756). There is no significant correlation between the standing long jump performance and the PSQI score (*β* = 0.000, 95% CI: −0.132 ∼ 0.132, *p* = 0.999). There is also no significant correlation between sit-and-reach performance and the PSQI score (*β* = −0.172, 95% CI: −0.595 ∼ 0.173, *p* = 0.274).

**Table 2:** Associations between physical fitness measures and sleep quality (PSQI score).

Predictor	*β*	95% CI	*p* value
Vital capacity (mL)	−0.139	−0.001 to 0.000	0.361
50-m sprint (s)	0.086	−3.044 to 4.165	0.756
Standing long jump (cm)	0	−0.132 to 0.132	0.999
Sit-and-reach (cm)	−0.172	−0.595 to 0.173	0.274

Values are standardized regression coefficients (β).

The 95% confidence intervals (CIs) are based on unstandardized coefficients (B).

All models were adjusted for age and sex.

### 3.3 Associations between physical fitness indicators and anxiety symptoms

The relationship between physical fitness indicators and anxiety symptoms evaluated by HADS-A scores is shown in Table 3. After adjusting age and gender, no significant correlation was found between the physical fitness indicators and the HADS-A score (all *p* > 0.05). Vital capacity is not related to anxiety symptoms (*β* = 0.001, 95% CI: −0.001 ∼ 0.001, *p* = 0.996). The 50-meter sprint score is positively but not significantly related to the HADS-A score (*β* = 0.372, 95% CI: −1.076 ∼ 5.735, *p* = 0.175). There is no significant correlation between standing long jump performance and anxiety symptoms (*β* = 0.168, 95% CI: −0.086 ∼ 0.163, *p* = 0.535). There is also no significant correlation between the anterior flexion performance of the sitting body and the HADS-A score (*β* = −0.090, 95% CI: −0.469 ∼ 0.256, *p* = 0.558).

**Table 3:** Associations between physical fitness measures and anxiety symptoms (HADS-A score).

Predictor	*β*	95% CI	*p* value
Vital capacity (mL)	0.001	−0.001 to 0.001	0.996
50-m sprint (s)	0.372	−1.076 to 5.735	0.175
Standing long jump (cm)	0.168	−0.086 to 0.163	0.535
Sit-and-reach (cm)	−0.090	−0.469 to 0.256	0.558

Values are standardized regression coefficients (β).

The 95% confidence intervals (CIs) are based on unstandardized coefficients (B).

All models were adjusted for age and sex.

### 3.4 Associations between physical fitness indicators and sleep disturbance

The relationship between physical fitness indicators and sleep sleep disturbance (PSQI > 5) is shown in Table 4. After adjusting age and gender, no significant correlation was found between various physical fitness indicators and sleep disturbance (all *p* > 0.05). There is no significant correlation between the lung capacity and the ratio of sleep disturbance (OR = 1.00, 95% CI: 1.00–1.00, *p* = 0.783). Similarly, there is no significant correlation between 50-meter sprint results and sleep disturbance (OR = 0.81, 95% CI: 0.28–2.33, *p* = 0.698). There is no significant correlation between standing long jump performance and sleep disturbance (OR = 0.99, 95% CI: 0.96–1.03, *p* = 0.728). There is also no significant correlation between the performance of sitting body flexion and sleep disturbance (OR = 0.92, 95% CI: 0.82–1.04, *p* = 0.193).

**Table 4:** Associations between physical fitness measures and sleep disturbance (PSQI > 5).

Predictor	OR	95% CI	*p* value
Vital capacity (mL)	1	1.00–1.00	0.783
50-m sprint (s)	0.81	0.28–2.33	0.698
Standing long jump (cm)	0.99	0.96–1.03	0.728
Sit-and-reach (cm)	0.92	0.82–1.04	0.193

OR, odds ratio. ORs are reported per 1-unit increase in each predictor (Vital capacity in mL, 50m-sprint time in s, standing long jump in cm and sit-and-reach in cm).

95% confidence intervals (CIs) were calculated based on Exp(B).

Models were adjusted for age and sex.

### 3.5 Associations between physical fitness indicators and symptoms of increased anxiety

The relationship between physical fitness indicators and increased anxiety symptoms (HADS-A ≥ 8) is shown in Table 5. After adjusting age and gender, no significant association was observed between various physical fitness indicators and symptoms of increased anxiety (all *p* > 0.05). There is no significant correlation between lung capacity and increased anxiety symptoms (OR = 1.00, 95% CI: 1.00–1.00, *p* = 0.743). There is no significant correlation between the 50-meter sprint performance and the symptoms of increased anxiety (OR = 1.88, 95% CI: 0.40–8.92, *p* = 0.425). There is no significant correlation between standing long jump and symptoms of increased anxiety (OR = 1.01, 95% CI: 0.96–1.06, *p* = 0.740). There is also no significant correlation between the performance of anterior flexion of the sitting body and the symptoms of increased anxiety (OR = 0.97, 95% CI: 0.81–1.15, *p* = 0.691).

**Table 5:** Associations between physical fitness measures and elevated anxiety symptoms (HADS-A ≥ 8).

Predictor	OR	95% CI	*p* value
Vital capacity (mL)	1.00	0.9998–1.0004	0.743
50-m sprint (s)	1.88	0.40–8.92	0.425
Standing long jump (cm)	1.01	0.96–1.06	0.74
Sit-and-reach (cm)	0.97	0.81–1.15	0.691

OR, odds ratio. ORs are reported per 1-unit increase in each predictor (Vital capacity in mL, 50m-sprint time in s, standing long jump in cm and sit-and-reach in cm).

95% confidence intervals (CIs) were calculated based on Exp(B).

Models were adjusted for age and sex.

## 4 Discussion

This study explores the correlation between the physical fitness components of a variety of objective evaluations and the sleep quality and anxiety outcome of undergraduates. Contrary to the general hypothesis, no significant correlation between physical fitness indicators and sleep quality or anxiety outcomes was found in this study, whether the outcome variable was analyzed as a continuous score or a classified risk indicator. The results show that in the group of non-clinical college students, the general physical fitness level evaluated based on the standardized physical fitness test may have limited explanatory power to explain the differences between sleep quality and anxiety.

### 4.1 Explanation that there is no significant correlation between physical fitness and sleep and anxiety outcomes

Although physical activity and physical ability are widely believed to be beneficial to mental health and sleep, the latest evidence shows that these associations are not consistent in different groups of people and situations (24, 25). In relatively healthy undergraduate groups, differences in general physical fitness levels may not be enough to form a detectable statistical correlation with sleep quality or anxiety symptoms. Recent observational studies of non-clinical youth groups have also reported that the relationship between overall physical fitness or activity level and mental health outcomes is weak, inconsistent or insignificant (26, 27).

It should be emphasized that the results of no significant correlation found in this study do not deny that structured exercise has a positive effect on sleep and anxiety as shown by interventional research (28). On the contrary, the results of this study highlight the important difference between exercise intervention and cross-sectional general physical status assessment. It suggests that based on physical fitness level alone, regardless of exercise behavior, exercise intensity or psychosocial situation, may not be enough to fully explain the sleep or anxiety outcome of college students.

The present findings can be further interpreted within an integrative lifestyle framework. Objective physical fitness primarily reflects physiological capacity (what an individual is capable of), whereas sleep quality and anxiety symptoms in university settings are often more proximally shaped by daily behaviors and contextual exposures (what an individual actually does and experiences), such as sedentary time, screen exposure, irregular schedules, and academic or psychosocial stress. Under this view, physical fitness may exert indirect effects on sleep and anxiety through behavioral engagement (e.g., habitual physical activity), time-use patterns across the day, and stress regulation, rather than showing strong direct cross-sectional associations with standardized fitness test performance. This “capacity versus behavior” distinction may help reconcile why structured exercise interventions can improve sleep or anxiety in controlled trials, while associations between fitness test indicators and mental health outcomes may appear weak or inconsistent in community-based undergraduate samples.

### 4.2 The contribution of objective physical fitness indicators to existing literature

Most studies on physical activity, sleep and mental health among young people rely on self-reported levels of physical activity, and such indicators are susceptible to memory bias and classification errors (29, 30). In contrast, this study adopts an objective and standardized physical fitness assessment, covering multiple fields of cardiopulmonary function, speed, muscle explosiveness and flexibility. Multiple physical fitness dimensions present consistent and no significant results, suggesting that objective physical indicators reflect physiological ability more than the level of daily behavioral participation, which may be more directly related to sleep quality and anxiety experience (31).

In addition, this study uses both continuous and two-classified outcomes to analyze sleep quality and anxiety, and obtains consistent results in different analysis methods. This consistency reduces the possibility that the research results will be affected by the outcome definition or model setting, which is an issue that is often concerned in mental health and behavior research (32).

### 4.3 Enlightenment on understanding the sleep and anxiety of college students

The results of this study emphasize that college students’ sleep quality and anxiety have multi-factorial characteristics. From a time-use perspective, sleep, sedentary behavior (including screen time), and physical activity co-occur within a finite 24-hour day, and changes in one domain may displace time in another. Previous studies have shown that academic stress, electronic screen exposure, irregular routine, emotional regulation ability and psychosocial stress play a central role in the sleep and mental health outcome of this group (33). Under this broader framework, general physical fitness may be just one of many influencing factors, and its impact may be relatively limited when examined alone.

From a practical point of view, this study suggests that interventions aimed at improving general physical fitness alone may not be enough to solve sleep problems or anxiety symptoms in non-clinical students. A comprehensive health promotion strategy that combines physical activity with stress management, sleep health education and psychological support may be more conducive to improving the physical and mental health of college students (34).

The relatively high mean PSQI and HADS-A scores observed in this non-clinical undergraduate sample warrant careful interpretation. After verifying the scoring procedures and data ranges, one plausible explanation is that elevated sleep disturbance and anxiety symptoms may be common in university settings, potentially reflecting academic pressure, irregular schedules, and psychosocial stressors. Importantly, this study did not conduct clinical diagnostic screening, the questionnaire scores should be interpreted as self-reported symptom levels rather than clinical diagnoses; therefore, elevated questionnaire scores can still occur. Nevertheless, the high prevalence underscores the need for cautious interpretation and replication in larger, multi-site samples.

### 4.4 Strengths and limitations

This study has the following advantages. First of all, the use of objective physical fitness indicators improves the effectiveness of the measurement, and reduces the bias of the report compared with the level of self-reporting activities. Secondly, sleep quality and anxiety outcomes are examined at the same time, and a more comprehensive evaluation of mental health-related outcomes is provided in combination with continuous and classification analysis methods. Third, transparent reports without significant results can help alleviate the bias of publication, which is still an important issue in the field of behavior and health science.

At the same time, it should be admitted that there are some limitations in this study. Cross-sectional research design limits causal inference, and longitudinal or interventional research is needed to clarify the chronological relationship between physical ability and mental health outcomes. Odds ratios derived from small samples with high outcome prevalence should be interpreted cautiously, as they may overestimate effect sizes. Due to the relatively small sample size and the high prevalence of the outcomes, the regression analyses should be interpreted with caution and should be considered exploratory in nature. Future studies with larger and more diverse samples are warranted to confirm the robustness and generalizability of these findings. In addition, the sample of this study comes from a single university, which may limit the extraportion of the results to other educational or cultural situations. Sedentary behavior and screen exposure were not directly assessed, which limits our ability to test broader lifestyle pathways that may link daily time use to sleep and anxiety. Cognitive or academic performance indicators were also not measured; therefore, the potential implications of sleep and anxiety for mental functioning in this sample cannot be evaluated.

## 5 Conclusion

In a word, this study found that in the undergraduate group, there is no significant correlation between the objectively measured physical fitness components and sleep quality or anxiety outcome. The research results suggest that general physical fitness may have a limited role in explaining the differences in sleep and anxiety of non-clinical college students. By providing well-founded and insignificant evidence based on objective physical fitness assessment, this study helps to deepen the understanding of the boundary between physical fitness and mental health, and emphasizes the importance of adopting a multi-dimensional integrated research paradigm in student health research.

## Data Availability

The raw data supporting the conclusions of this article will be made available by the corresponding author upon reasonable request.
